# Psychomedial Clairvoyant

**Published:** 1854-11

**Authors:** 


"*Psychomedical Clairvoyant.*"—The following advertisement we clip from the "Hartford (Conn.) Daily Courant," bearing date Oct. 9, 1854. As all of your readers may not be posted in the newest modes of medical practice and diagnostic requirements preliminary, we append it as latest and best from Connecticut. What the need of so much *hair* we are at a loss to surmise, unless, noting the partner Swan, the Madame wishes to nest, in words technical, it is sought for *clinical* purposes! W. T.

"*Psychomedical Clairvoyance.*—Dr. Swan and Madame Johnson, the well known and successful Mesmeric and Botanic Physicians, are permanently located at 41 College street, Hartford, Ct., where they will be happy to wait upon all who are disposed to avail themselves of their superior skill in clairvoyant practice, on all diseases that the human system is heir to. Satisfaction guaranteed or no pay. *Terms*—Examination of disease and prescriptions, personally, $1; Examinations by letter, enclosing a lock of hair of the patient, $3 in advance."

